# Elective breast radiotherapy including level I and II lymph nodes: A planning study with the humeral head as planning risk volume

**DOI:** 10.1186/s13014-016-0759-7

**Published:** 2017-01-18

**Authors:** Kathrin Surmann, Jorien van der Leer, Tammy Branje, Maurice van der Sangen, Maarten van Lieshout, Coen W. Hurkmans

**Affiliations:** Department of Radiation Oncology, Catharina Hospital, Michelangelolaan 2, 5623EJ Eindhoven, The Netherlands

**Keywords:** Breast radiotherapy, Lymph nodes, IMRT, VMAT, Humeral head, Planning study

## Abstract

**Background:**

The aim of this study was to assess the dose to the humeral head planning risk volume with the currently used high tangential fields (HTF) and compare different planning techniques for breast radiotherapy including axillary level I and II lymph nodes (PTVn) while sparing the humeral head.

**Methods:**

Ten patients with left-sided breast cancer were enrolled in a planning study with 16 fractions of 2.66 Gy. Four planning techniques were compared: HTF, HTF with sparing of the humeral head, 6-field IMRT with sparing of the humeral head and VMAT with sparing of the humeral head. The humeral head + 10 mm was spared by restricting V40Gy < 1 cc.

**Results:**

The dose to the humeral head was too high with HTF (V40Gy on average 20.7 cc). When sparing the humeral head in HTF, PTVn V90% decreased significantly from 97.9% to 89.4%. 6-field IMRT and VMAT had a PTVn V90% of 98.2% and 99.5% respectively. However, dose to the lungs, heart and especially the contralateral breast increased with VMAT.

**Conclusions:**

The humeral head is rarely spared when using HTF. When sparing the humeral head, the 6-field IMRT technique leads to adequate PTV coverage while not increasing the dose to the OARs.

## Background

In case of limited metastatic sentinel node involvement the choice between axillary dissection or breast irradiation with or without axillary radiotherapy does not seem to make much difference in terms of regional relapse and survival. Both treatment modalities lead to sporadic regional recurrences [1, 2]. Irradiated volume and toxicity are related and this relationship plays a role in the choice of treatment.

Several studies reported decreased mobility of the shoulder after radiotherapy [1, 3–6]. The AMAROS trial compared radiotherapy and surgery of the axilla for patients with a positive sentinel lymph node [1]. Both groups reported decreased arm mobility with a worse quality of life for patients that underwent radiotherapy treatment compared to patients with an axillary lymph node dissection. It is suggested that irradiation of the shoulder tissue leads to decreased mobility. The ESTRO consensus guideline for target delineation for elective breast and nodal radiotherapy establishes the humeral head and connective tissues 10 mm around it as planning risk volume (PRV) [7].

Tangential fields are widely used for breast radiotherapy [8–11]. Modified high tangential fields (HTF) have been proposed to ensure coverage of the level I and II lymph nodes [12, 13]. Ohashi et al. extended the tangential fields in the cranial and posterior direction to include level I—III lymph nodes [13], while Alco et al. extended the cranial and posterior border to include level I and II lymph nodes [12]. IMRT techniques for breast radiotherapy emerged over the past years due to the improved dose homogeneity and target coverage compared to HTF [9, 14–16]. Dogan et al. concluded that sparing of the humeral head while maintaining target coverage is best achieved with a 9-field IMRT technique for the whole breast with internal mammary nodes, supraclavicular and axillary lymph nodes [14]. More recently, VMAT techniques have been explored for breast radiotherapy due to the improved dose homogeneity and target coverage compared to HTF and IMRT for whole breast radiotherapy. While reducing hotspots (dose > 107%) in the PTV and high doses in organs at risk (OAR), larger low dose regions in OARs have been reported [15, 17–19]. Osman et al. found improved target coverage, conformity and reduced lung doses when comparing VMAT with 3D conformal radiotherapy plans for the breast, internal mammary node and periclavicular lymph nodes [15]. However, the average dose to the contralateral breast increased when using VMAT.

The aim of this planning study was to assess the dose to the humeral head PRV with the currently used HTF. If this dose is too high, the influence of the humeral head sparing on the coverage of target volumes with HTF is determined. Finally, the optimal technique for humeral head sparing in elective breast radiotherapy including level I and II lymph nodes will be determined by comparing different planning techniques.

## Methods

### Patients and equipment

Ten consecutive patients with left-sided breast cancer previously treated at our institute were enrolled in a planning study performed in Pinnacle^3^ (v9.8; Philips). This patient dataset reflects the variations in the irradiated anatomy in our patient population well. Treatment planning of left-sided breast radiotherapy is more challenging due to the proximity of the heart to the treatment volume and increased heart dose compared to right-sided breast radiotherapy. Voluntary breath hold is state of the art for left-sided breast radiotherapy to reduce the heart dose [20] and was used during treatment of all ten patients.

**Fig. 1:**
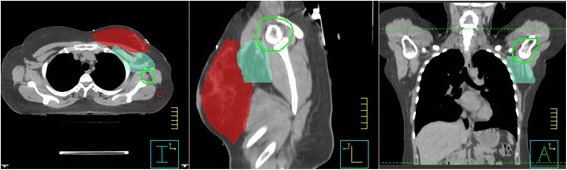
Axial, sagittal and coronal slices of the planning CT of patient 2. PTVp (primary) in *red*, PTVn (level I and II lymph nodes) in *turquoise* and humeral head + 10 mm in *green*

The planning CT was acquired with a Big Bore CT scanner (Philips, Best, The Netherlands) and a slice spacing of 3 mm. Patients were scanned on a breast board with both arms resting in an arm support above the head. CTV and GTV were contoured by the radiation oncologist according to the ESTRO guidelines [7] and expanded with a 7 mm PTV margin while excluding the skin with a margin of 7 mm and the lungs without an additional margin (see Fig. 1). The heart was contoured according to Feng et al. [21].

### Treatment planning techniques

In this planning study, we compared four techniques:

- High tangential field (HTF)
- HTF with sparing of the humeral head
- 6-field IMRT with sparing of the humeral head
- VMAT with sparing of the humeral head

The humeral head PRV (hh+10) was spared by restricting V40Gy < 1 cc for the last three techniques. Data on the critical dose the humeral head is scarce and we chose to follow the recommendation of Dogan et al. who employ a maximum dose of 40 Gy for the humeral head in their planning study [14]. Treatment plans were obtained with the inverse planning tool and optimization was achieved by decreasing the dose to the OARs (lungs, heart and contralateral breast) as low as possible while maintaining a PTVp (breast) V95% ≥ 97% and PTVn (level I and II lymph nodes) V90% ≥ 95%. V107% was limited to 2% of the target volume. The initial set of planning objectives is given in Table 1.

**Table 1:** Initial set of objectives used for the inverse optimization

Region of Interest	Type	Target (cGy)	Volume (%)	Weight	gEUD
PTVp-Lungs-Skin07^a^	Min DVH	4043	99	75	
	Max DVH	4470	1	1	
	Uniform Dose	4256		1	
PTVn-Lungs-Skin07	Min DVH	4043	99	75	
	Max DVH	4430	1	1	
	Uniform Dose	4256		0.1	
15_Ringtot_30^b^	Max Dose	4256		5	
Lungs	Max EUD	700		1	1
Heart	Max EUD	700		1	1
For 6-field IMRT and VMAT with sparing of the humeral head:					
10_Ring_10^c^	Max Dose	3900		1	
hh+10^d^	Max Dose	3800		1	

^a^PTVp excluding the lungs and the skin + 7 mm

^b^A ring at 15 mm distance from PTVtotal with a width of 30 mm, excluding the lungs and skin + 7 mm

^c^A ring at 10 mm distance from PTVtotal with a width of 10 mm, with the caudal border 1 cm below the sternoclavicular joint and excluding the skin + 7 mm

^d^Humeral head + 10 mm

All techniques were planned with 6 MV photon beams. For the HTF, the cranial and posterior border of the tangential fields was extended to include PTVn. The maximum number of segments was 8 with a minimum area of 9 cm^2^ and a minimum of 4 MU per segment. The collimator angle was 0 degree. These setting were the same for HTF with and without sparing of the humeral head. The plan was optimized with the initial objectives of Table 1 to fulfil the previously mentioned evaluation criteria for PTV coverage and minimized OAR doses. For HTF with sparing of the humeral head, the leaves of the 10 mm MLC were then manually closed to exclude hh+10 and reduce the dose to the humeral head and surrounding tissue.

**Fig. 2:**
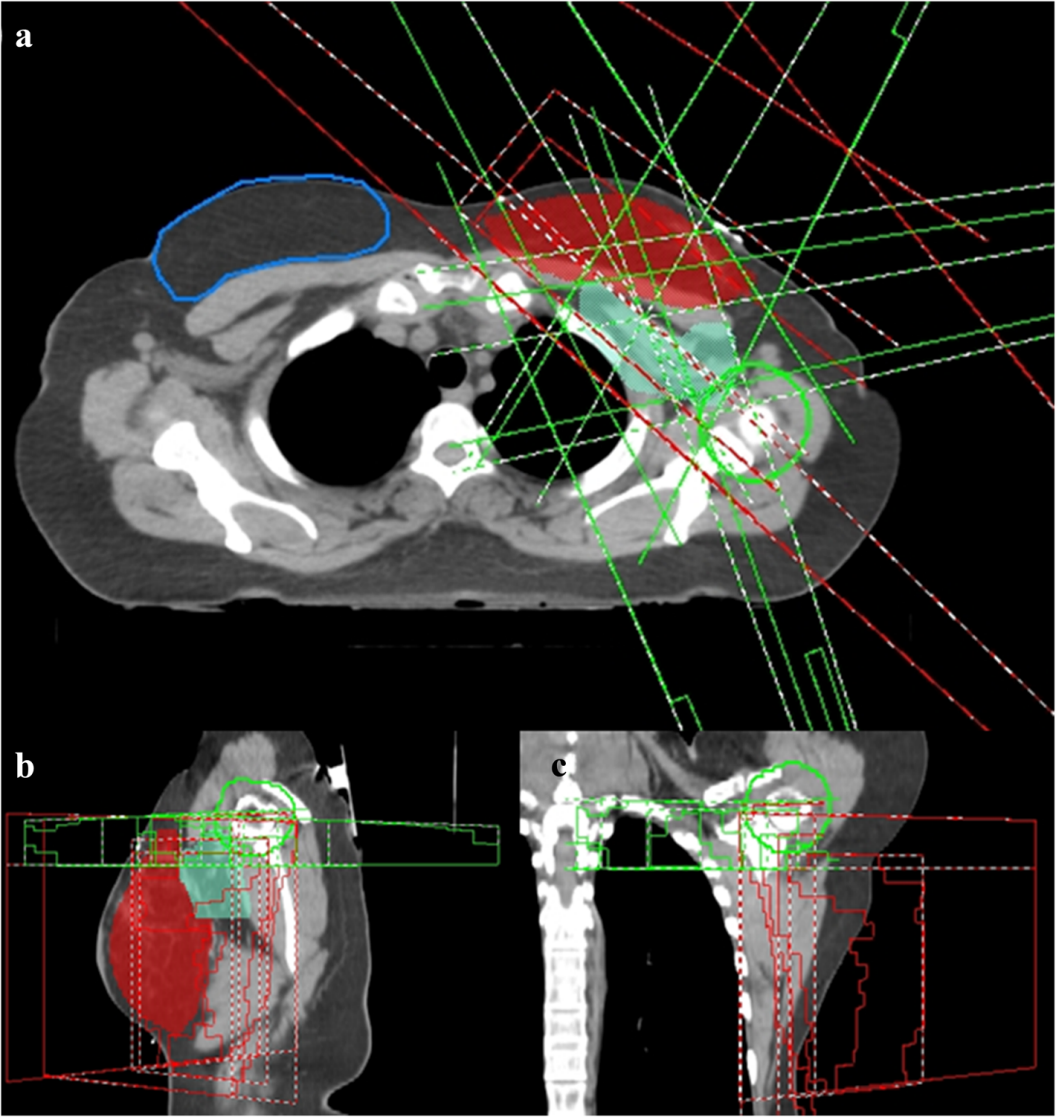
Beam arrangement for the 6-field IMRT technique; **a** axial, **b** sagittal and **c** coronal view. Two high tangential fields (*red*) and four additional fields (*green*). PTVp in *red*, PTVn in *turquoise*, contralateral breast in *blue* and humeral head + 10 mm in *green*

The 6-field IMRT technique consisted of two high tangential fields and four additional fields (at around 330, 20, 80 and 170 degrees, see Fig. 2) to ensure proper coverage of the cranial part of the breast and the lymph nodes. The two tangential fields were the same fields as in the HTF planning without sparing of the humeral head and administered 60% of the MU. The caudal border of the four additional fields was set 1 cm below the sternoclavicular joint and the isocentre was placed in that slice. The minimal craniocaudal dimension of the additional fields was 3 cm. The maximum number of segments was 20 with a minimum area of 9 cm^2^ and a minimum of 4 MU per segment. The collimator angles of the high tangential and additional fields were 0 and 90 degree, respectively.

The fourth technique was a VMAT dual arc from around 310 to 180 degrees with control points every 4 degrees. In the optimization parameters, the maximum delivery time was 60 s per arc. The minimum segment area was 6 cm^2^ and the collimator was rotated 10 degree to minimize tongue-and-groove effects.

### Plan evaluation

Dose parameters were recorded for each plan. V90% and V95% of PTVp and PTVn were obtained, as well as the average dose to the lungs, heart, the contralateral breast and the V40Gy of hh+10. The conformity indices CI95% and CI90% were calculated as the ratio between the volume of the 95% or 90% isodose and the total PTV. Dose parameters of two planning techniques were tested for statistical significance with a paired two-tailed Wilcoxon signed rank sum test in Excel. It was corrected for multiple testing with the Bonferroni method (*n* = 4) and differences were considered significant when *p* ≤ 0.0125. In addition, the volume overlap between hh+10 and the PTVtotal was measured. In a sub-analysis, it was investigated whether the overlap between these two structures could indicate which patient benefits from a humeral head sparing technique and which could be treated properly with the current standard of HTF (see Appendix).

## Results

HTF resulted in an average PTVp V95% of 97.8% and an average PTVn V90% of 97.9% (Table 2). When sparing the humeral head, this decreased to 97.2% (*p* = 0.02 compared to HTF) and 89.4% (*p* ≤ 0.01 compared to HTF), respectively. With the additional fields of the 6-field IMRT technique, the coverage of the lymph nodes increased significantly to on average 98.2% (*p* ≤ 0.01 compared to HTF hh) while PTVp did not vary significantly (*p* = 0.65 compared to HTF hh). The doses to the OAR were comparable between the HTF without and with sparing of the humeral head and the 6-field IMRT technique (Table 2). The coverage of PTVn increased when using VMAT to an average of 99.5% (*p* = 0.51 compared to IMRT and *p* ≤ 0.01 compared to HTF hh). The conformity indices improved significantly for each change in technique (Table 2). Note that CI were calculated for the total PTV, combining PTVp and PTVn. However, the dose to the OAR increased as well when using the VMAT technique. The mean dose to the contralateral breast increased significantly from 0.7 Gy with HTF and 0.6 Gy with HTF hh and 6-field IMRT to 2.3 Gy with VMAT (*p* ≤ 0.01 compared to IMRT and HTF hh).

**Table 2:** Dose parameters for the PTVs and OARs for all four techniques

	HTF	HTF – HTF hh *p*-value	HTF hh	HTF hh – IMRT *p*-value	IMRT	IMRT – VMAT *p*-value	VMAT	HTF hh – VMAT *p*-value
PTVp V90% (%)	99.9 (99.7–100)	**≤0.01**	99.5 (95.6–100)	0.08	99.8 (99.2–100)	**≤0.01**	99.5 (98.8–99.9)	0.08
PTVp V95% (%)	97.8 (97.2–99.5)	0.02	97.2 (91.8–99.5)	0.65	97.3 (96.7–98.8)	0.09	97.1 (96.6–97.4)	0.14
PTVn V90% (%)	97.9 (96.6–99.3)	**≤0.01**	89.4 (73.3–98.7)	**≤0.01**	98.2 (91.3–99.9)	0.51	99.5 (98.2–99.9)	**≤0.01**
PTVn V95% (%)	86.5 (81.4–94.2)	**≤0.01**	73.6 (56.9–87.6)	**≤0.01**	91.1 (77.8–98.2)	0.05	95.4 (88.3–98.3)	**≤0.01**
CI95%	1.24 (1.04–1.41)	**≤0.01**	1.16 (1.01–1.29)	**≤0.01**	1.06 (0.93–1.18)	**≤0.01**	0.90 (0.77–1.06)	**≤0.01**
CI90%	1.49 (1.25–1.66)	**≤0.01**	1.41 (1.22–1.51)	**≤0.01**	1.33 (1.14–1.41)	**≤0.01**	1.09 (0.97–1.25)	**≤0.01**
Lungs Dmean (Gy)	4.8 (3.9–6.1)	0.02	4.7 (3.9–6.1)	0.33	4.8 (3.8–5.9)	0.24	5.2 (4.2–6.8)	0.17
Heart Dmean (Gy)	3.3 (1.7–6.2)	**≤0.01**	3.3 (1.6–6.1)	**≤0.01**	2.9 (1.6–5.7)	0.04	3.6 (2.0–5.7)	0.51
Contralat. breast Dmean (Gy)	0.7 (0.3–0.9)	**≤0.01**	0.6 (0.3–0.9)	0.02	0.6 (0.3–0.7)	**≤0.01**	2.3 (0.6–4.2)	**≤0.01**
hh+10 V40Gy (cc)	20.7 (0–66.9)	**≤0.01**	0.5 (0–1.2)	0.37	0.6 (0–1.8)	0.31	0.6 (0–1.3)	0.26

*HTF* High tangential fields, *HTF hh* HTF with sparing of the humeral head, *CI95%* Conformity index of the 95% isodose, *CI90%* Conformity index of the 90% isodose, *hh+10* humeral head + 10 mm. Values are averages with the range indicated in brackets. *p*-values are written in bold when statistical significance is reached (*p* ≤ 0.0125)

A single patient out of ten met the constraint for hh+10 with HTF. This patient was also the only patient that did not have an overlap between hh+10 and the total PTV. The overlap was on average 2.1 cc (0–5.1 cc).

**Fig. 3:**
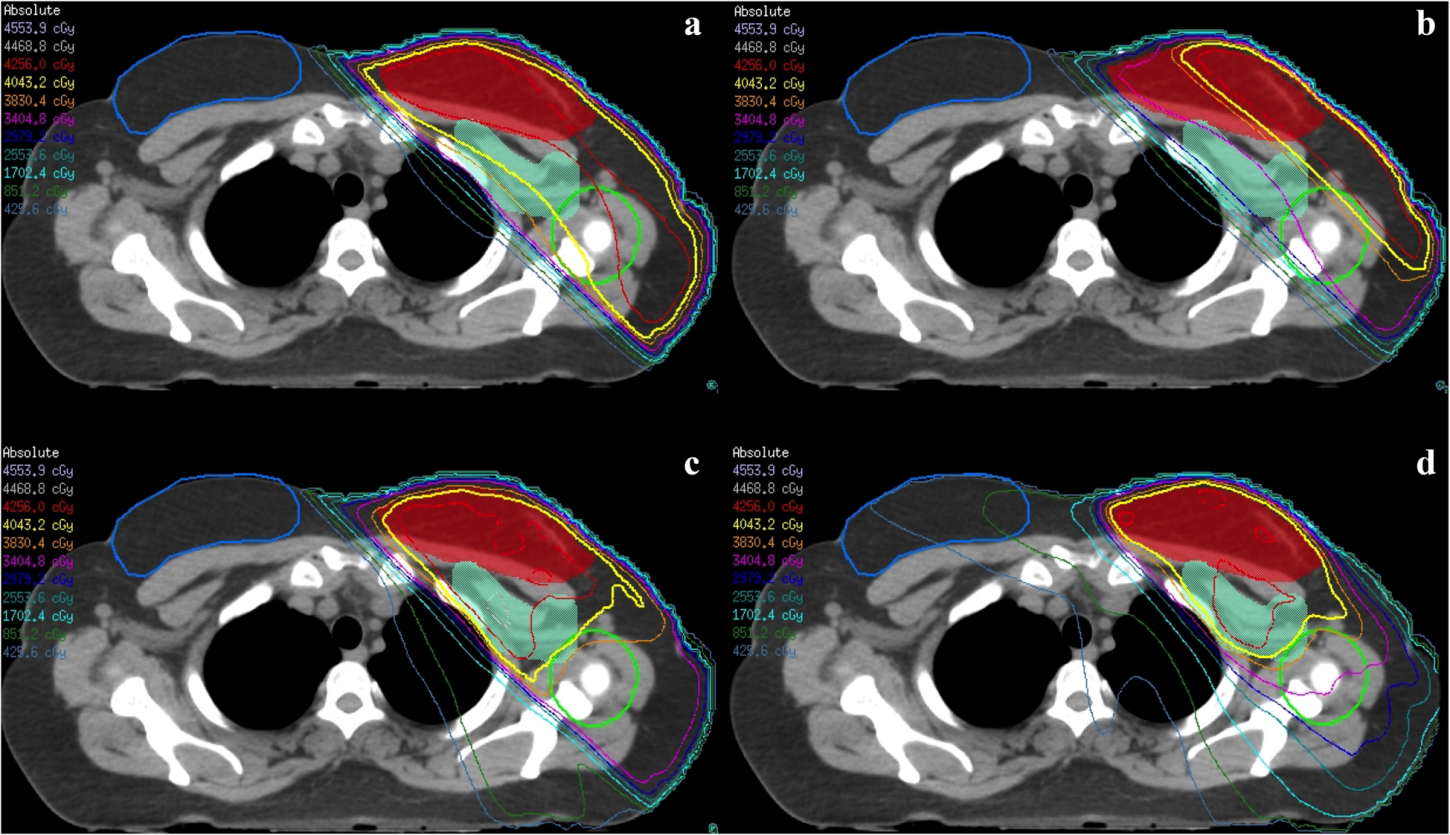
Dose distributions of **a** HTF, **b** HTF with sparing of the humeral head, **c** 6-field IMRT with sparing of the humeral head and **d** VMAT with sparing of the humeral head. PTVp in *red*, PTVn in *turquoise*, contralateral breast in *blue* and humeral head + 10 mm in *green*. Isodose lines: *red*: 100%, *yellow*: 95% and *orange*: 90%

Examples of the dose distributions of all four techniques can be found in Fig. 3.

## Discussion

In case of elective breast radiotherapy including the axillary levels I and II, we compared four planning techniques and evaluated the coverage of the PTVs and the OARs. We found that 6-field IMRT with sparing of the humeral head resulted in the best coverage of PTVp and PTVn while not increasing the dose to the lungs, heart and contralateral breast compared to HTF.

The coverage of PTVn was increased significantly with the 6-field IMRT technique compared to the HTF. Dogan et al. treated the whole breast and the internal mammary node, supraclavicular and axillary lymph nodes with a 6-field IMRT technique and achieved a D95% of 50.2 ± 0.8 Gy to the axillary lymph nodes with a prescription dose of 50 Gy in 25 fractions [14]. With a 2-field IMRT approach similar to our HTF, this was 49.8 ± 0.8 Gy. However, sparing of the humeral head was not achieved with these techniques. In our study, the most optimal sparing of the humeral head was achieved with the 6-field IMRT, which is similar to their 6-field IMRT technique, and VMAT technique while still meeting the constraints for lymph node coverage. Dogan et al. mentioned treatment complexity as one of the factors for choosing 2- and 4-field IMRT as optimal techniques. Currently, treatment complexity (i.e. number of IMRT beams) is no longer a limiting factor.

Alco et al. recorded a V95% of 94.4% (85.3–98.7%) in level I and 90.1% (80.6–94.4%) in level II lymph nodes with modified HTF [12]. The tangential fields were modified to extend just until the inferior border of the humeral head and the MLCs were manipulated to include the level I and II lymph nodes. Alco et al. did not spare the humeral head. We found a lower PTVn V95% with the two HTF techniques, but when using 6-field IMRT and VMAT the V95% improved. With the more advanced 6-field IMRT and VMAT technique including sparing of the humeral head, our PTVn V95% was comparable to Alco et al. with IMRT and better than Alco et al. with VMAT.

Ohashi et al. reported level I and II V90% of 97.6% (96.0–99.2%) and 89.5% (84.2–93.2%) with modified HTF [13]. Their HTF technique was similar to Alco et al. and did also not spare the humeral head. In our study, PTVn coverage with HTF is higher (average V90% 97.9%, range 96.6–99.3), but decreased to an inacceptable level when sparing the humeral head. Again, the more advanced IMRT and VMAT techniques reached the same or better coverage compared to Ohashi et al. while sparing the humeral head.

Belkacemi et al. concluded that HTF did not result in adequate coverage of the level I and II lymph nodes [11]. They extended the cranial border of the tangential fields until the inferior border of the humeral head similar to Ohashi et al. and Alco et al., but did not extend the posterior field border. In our experience, the cranial and posterior part of the lymph nodes can extend beyond the caudal border of the humeral head in the tangential fields and therefore be outside the treatment field. In our study, the cranial and posterior border of the HTF was based on the dimensions of PTVn and resulted in sufficient PTVn coverage. PTVn coverage decreased significantly when sparing the humeral head by closing the MLC due to the overlap between PTV and hh+10; particularly a too low dose in level II (see Table 2 and Fig. 3b).

Dogan et al. concluded that high doses in the heart can be reduced with IMRT techniques while maintaining excellent coverage of the breast and regional nodes [14]. Schubert et al. [9] compared different planning techniques for the whole breast and reported the following OAR dose parameters with an inverse IMRT technique: Dmean heart 1.9 ± 0.8 Gy and Dmean contralateral breast 0.3 ± 0.1 Gy. With tomotherapy, these parameters were 3.9 ± 1.3 Gy and 0.6 ± 0.1 Gy, respectively. We found slightly larger average doses to the heart and contralateral breast (Table 2) due to the increased treatment volume when axillary lymph nodes are involved. When comparing the four techniques, the differences between the mean dose to the heart and contralateral breast are often statistically significant. However, the differences are very likely in most cases not clinically relevant except for the increase in contralateral breast dose when comparing IMRT and VMAT. Osman et al. also found a similar increase in contralateral breast dose when using VMAT (Dmean on average 2.7 Gy) compared to forward planned IMRT (Dmean on average 0.7 Gy) when treating the breast, internal mammary node and periclavicular lymph nodes [15].

When comparing these planning studies, it has to be noted that lymph node contouring may have been based on different contouring guidelines. The ESTRO guideline by Offersen et al. [7] focusses on the delineation of level I—IV lymph nodes and has been used in this planning study. In addition, contouring is prone to variabilities and even when the same guideline is followed, its interpretation can vary [22]. A PRV of the humeral head with a margin of 10 mm is defined by Offersen et al. [7], but no dose constraints are indicated.

Data on the dose to the humeral head and surrounding tissue during breast radiotherapy is scarce. In our planning study we restricted the high dose to hh+10. Dogan et al. based their constraint of a maximum dose of 40 Gy on their clinical experience with head and neck cancer [14].

As there is no consensus yet which DVH parameters for the humeral head PRV are clinically most relevant, we have also recorded Dmin, Dmean, Dmax, D0.5cc, D2%, D98% and V50% (see Appendix Table 3). Ideally, this constraint should be based on data correlating the change in shoulder mobility to the dose in the shoulder tissue. To the best of our knowledge, there is no literature on a dose-effect relationship for the shoulder tissue and the shoulder mobility. Delineation and dose assessment of the shoulder muscles should be further investigated but is outside the scope of this article.

## Conclusion

The humeral head is rarely spared when using conventional high tangential fields. It is not possible to spare the humeral head with HTF without reducing lymph node coverage of level I and II. The humeral head and surrounding tissues can be spared with the 6-field IMRT and VMAT technique without reducing PTVn coverage. The 6-field IMRT technique does not increase the dose to the OARs compared to HTF, while the VMAT technique leads to a higher dose in the contralateral breast.

## Figures and Tables

**Fig. 4:**
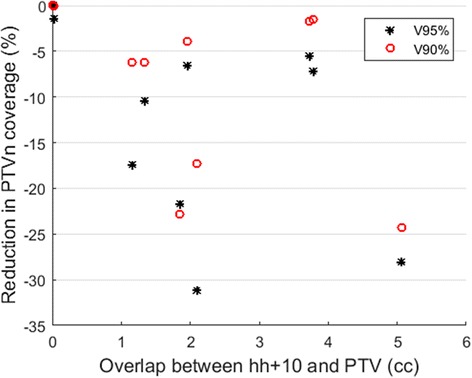
Reduction of PTVn coverage (V90% and V95%) due to sparing of the humeral head when using high tangential fields

## Appendix

### Influence of overlap between PRV and PTV on nodal PTV coverage

We started a sub-analysis in which we investigated whether it is possible to indicate which patients will benefit from a more advanced technique and which patients would be treated properly with the standard of high tangential fields. A more advanced technique with multiple beams takes longer to plan and requires a larger time slot at the treatment machine for delivery.

We determined the overlap between the total PTV and the humeral head + 10 mm PRV (hh+10) in cc and the reduction of PTVn coverage between HTF with and without sparing of the humeral head.

The results of this sub-analysis are given in Fig. 4. The overlap between PTVn and hh+10 was on average 2.1 cc (0–5.1 cc). Sparing the humeral head by closing the MLC in the HTF reduced PTVn V95% by 12.9% (0–31.2%) and PTVn V90% by 8.4% (0–24.3%). It was expected that a larger overlap between the PTV and hh+10 will result in a large reduction of PTVn coverage since excluding hh+10 from the tangential treatment fields will also mean exclusion of the overlapping PTV. However, there was no correlation between the volume overlap of the PTV and hh+10 and the reduction in PTVn dose between HTF and HTF hh (Fig. 4).

Based on the ten patients from our planning study, it is not possible to determine a maximum value for the overlap that will still result in proper treatment with HTF while sparing the humeral head. All patients will be treated with the more advanced technique to spare the humeral head and maintain proper PTVn coverage.

### Additional dose parameters for the humeral head + 10 mm PRV

The additional dose parameters that were scored for the humeral head + 10 mm PRV can be found in Table 3.

**Table 3:** Dose parameters for the humeral head + 10 mm PRV

	HTF	HTF – HTF hh *p*-value	HTF hh	HTF hh – IMRT *p*-value	IMRT	IMRT – VMAT *p*-value	VMAT	HTF hh – VMAT *p*-value
Dmin (Gy)	1.6 (0.8–3.2)	**≤0.01**	1.4 (0.8–2.1)	**0.01**	1.1 (0.5–1.8)	0.21	1.1 (0.4–1.9)	0.08
Dmean (Gy)	14.8 (3.8–27.8)	**≤0.01**	10.1 (3.8–15.5)	0.80	10.8 (2.8–19.7)	0.41	11.5 (2.6–19.9)	0.24
Dmax (Gy)	42.7 (38.2–44.6)	**≤0.01**	40.3 (38.2–41.9)	0.24	40.8 (38.5–42.3)	0.28	40.6 (38.1–42.2)	0.38
D0.5cc (Gy)	42.2 (36.8–43.9)	**≤0.01**	39.0 (34.6–40.7)	0.72	39.6 (36.2–41.2)	0.20	39.2 (34.8–40.7)	0.44
D2% (Gy)	40.9 (29.8–43.5)	**≤0.01**	36.2 (29.8–39.6)	0.26	37.3 (28.8–39.4)	0.17	36.9 (26.6–39.8)	0.92
D98% (Gy)	1.9 (0.9–3.7)	**≤0.01**	1.5 (0.8–2.3)	**≤0.01**	1.2 (0.5–2.1)	0.16	1.2 (0.5–2.1)	**≤0.01**
V50% (%)	28.6 (3.4–59.9)	**≤0.01**	15.6 (3.4–35.4)	0.11	23.8 (3.1–47.2)	0.38	25.9 (2.4–50.3)	0.08

*HTF* High tangential fields, *HTF hh* HTF with sparing of the humeral head. Values are averages with the range indicated in brackets. *p*-values are written in bold when statistical significance is reached (*p* ≤ 0.0125)

## Abbreviations

hh+10: Humeral head + 10 mm
HTF: High tangential fields
OAR: Organs at risk
PRV: Planning risk volume
PTVn: Planning target volume of the level I and II lymph nodes
PTVp: Planning target volume of the breast
